# Supplementary material for: Hot Water Treatment Causes Lasting Alteration to the Grapevine (Vitis vinifera L.) Mycobiome and Reduces Pathogenic Species Causing Grapevine Trunk Diseases
Source: J Fungi (Basel). 2022 May 6;8(5):485. doi: 10.3390/jof8050485 (PMC9144887; doi:10.3390/jof8050485)
Supplement: Supplementary file 1 [file jof-08-00485-s001.zip › jof-1689779-supplementary.pdf]

Supplementary Materials

Table S1. Contribution (r^2) of nursery, variety and treatment to variation in chlorophyll in graft unions. Annotation next to the numbers indicates significance level: ** < 0.01, n.s. = not significant (>0.05).

Factor	Graft Unions (r^2)
Nursery	0.089 **
Variety	0.028 n.s.
Treatment	0.000 n.s.
Full model	0.13 n.s.

Table S2. Indicator species for control and HWT plants in **(a)** graft union and **(b)** root collars for all OTUs. Graft unions and root collars were analyzed separately by variety and rootstock, respectively. Correlation values (stat) and the statistical significance of the correlation (p-values) are reported. GTD-related indicator species are marked with a cross (†) and biocontrol species are marked with an asterisk (*).

a. Graft unions: control vs HWT

50 species associated with control plants			
**Indicator Species**	**stat**	***p* value**	**significance**
Acaromyces ingoldii	0.58	0.001	***
Acrocalymma sp._3	0.26	0.03	*
Bionectria byssicola_1	0.56	0.011	*
Cadophora luteo-olivacea †	0.826	0.001	***
Cadophora luteo-olivacea †	0.387	0.003	**
Cadophora sp. †	0.767	0.001	***
Cadophora sp._1 †	0.368	0.001	***
Calloria sp.	0.449	0.002	**
Clonostachys sp.	0.483	0.026	*
Cortinarius sp.	0.424	0.046	*
Cystobasidium sp._2	0.26	0.029	*
Devriesia sp._1	0.309	0.015	*
Diaporthe sp._1 †	0.654	0.001	***
Diaporthe sp._2 †	0.26	0.03	*
Hannaella luteola	0.385	0.003	**
Leptosphaeria sp._5	0.778	0.005	**
Lophiostoma sp.	0.801	0.002	**
Lophiostoma sp._15	0.26	0.027	*
Lophiostoma sp._4	0.329	0.002	**
Lophiostoma sp._6	0.384	0.014	*
Meira nashicola	0.26	0.031	*
Meira sp.	0.553	0.004	**
Mycoarthris corallina	0.337	0.007	**

Niesslia sp._*4*	0.666	0.001	***
Niesslia sp._*5*	0.377	0.021	*
Occultifur sp.	0.26	0.033	*
Ophiobolus sp.	0.56	0.001	***
Pyrenochaeta sp.	0.458	0.038	*
Rosellinia sp.	0.359	0.011	*
Rosellinia sp._*1*	0.285	0.012	*
Sarocladium strictum_1	0.54	0.05	*
Sebacina sp.	0.259	0.045	*
Stagonospora sp._*4*	0.572	0.008	**
unknown_109	0.329	0.005	**
unknown_117	0.462	0.001	***
unknown_121	0.329	0.005	**
unknown_17	0.432	0.005	**
unknown_30	0.406	0.001	***
unknown_31	0.26	0.03	*
unknown_32	0.34	0.015	*
unknown_33	0.444	0.005	**
unknown_39	0.535	0.001	***
unknown_53	0.546	0.004	**
unknown_61	0.349	0.026	*
unknown_77	0.251	0.039	*
unknown_84	0.338	0.011	*
unknown_88	0.377	0.015	*
unknown_9	0.285	0.014	*
unknown_98	0.582	0.008	**
Vishniacozyma sp._*2* *	0.26	0.03	*
**38 species associated with HWT plants**			
**Indicator species**	**stat**	***p* value**	**significance**
Acremonium sp._*1* †	0.648	0.007	**
Acremonium sp._*12* †	0.426	0.024	*
Acremonium sp._*13* †	0.786	0.001	***
Acremonium sp._*14* †	0.658	0.001	***
Acremonium sp._*15* †	0.724	0.001	***
Acremonium sp._*5* †	0.401	0.013	*
Articulospora sp.	0.424	0.002	**
Cystobasidium laryngis	0.55	0.001	***
Cystobasidium lysinophilum	0.762	0.001	***
Cystobasidium slooffiae_1	0.695	0.001	***
Cystobasidium sp.	0.329	0.047	*
Entyloma sp.	0.557	0.031	*
Entyloma sp._*1*	0.503	0.044	*
Fusarium sp._*10*	0.4	0.002	**
Fusarium sp._*12*	0.315	0.017	*
Fusarium sp._*20*	0.527	0.001	***
Fusarium sp._*22*	0.786	0.004	**

Fusarium sp._*3*	0.823	0.001	***
Fusarium sp._*5*	0.423	0.04	*
Fusarium sp._*6*	0.689	0.001	***
Fusarium sp._*9*	0.283	0.026	*
Microstroma sp.	0.415	0.017	*
Nectria sp._*1* †	0.367	0.037	*
Phaeosphaeria sp._*3*	0.367	0.04	*
Phoma sp. †	0.417	0.022	*
Phoma sp._*1* †	0.358	0.021	*
Quambalaria sp.	0.342	0.026	*
Sporisorium sp.	0.414	0.003	**
Stagonospora sp.	0.526	0.004	**
Stagonospora sp._*1*	0.499	0.024	*
Stagonospora sp._*5*	0.671	0.001	***
Unknown	0.473	0.001	***
unknown_1	0.32	0.015	*
unknown_106	0.416	0.001	***
unknown_11	0.443	0.004	**
unknown_22	0.416	0.001	***
unknown_57	0.572	0.001	***
unknown_70	0.478	0.029	*

b. Root collars: control vs HWT

**23 species associated with control plants**			
**Indicator species**	**stat**	***p* value**	**significance**
Acaromyces ingoldii	0.494	0.0213	*
Bionectria byssicola	0.698	0.0409	*
Bionectria byssicola_1	0.315	0.0451	*
Cercophora samala	0.309	0.0285	*
Cystofilobasidium macerans	0.603	0.0324	*
Exophiala sp._*1*	0.603	0.0002	***
Leptosphaeria sp._*5*	0.761	0.0063	**
Meira sp._*1*	0.296	0.025	*
Ophiobolus sp.	0.504	0.0094	**
Phaeomoniella chlamydospora_3 †	0.715	0.0293	*
Phaeomoniella chlamydospora_4 †	0.327	0.0052	**
Phaeomoniella chlamydospora_5 †	0.322	0.0096	**
Phaeomoniella sp. †	0.359	0.0163	*
Phaeosphaeria sp._*4*	0.375	0.0419	*
Rhizoctonia sp.	0.551	0.0001	***
Serendipita sp.	0.567	0.0122	*
Stagonospora sp._*4*	0.551	0.0106	*
Thysanorea asiatica	0.487	0.0001	***
unknown_101	0.497	0.0003	***

unknown_108	0.306	0.0142	*
unknown_3	0.353	0.0472	*
unknown_79	0.283	0.0296	*
unknown_89	0.323	0.0208	*

29 species associated with HWT plants

**Indicator species**	**stat**	***p* value**	**significance**
Cercophora sp._*1*	0.264	0.0255	*
Chaetomium sp._*12*	0.513	0.0056	**
Cylindrocarpon sp._*4* †	0.51	0.0467	*
Cystobasidium sp._*2*	0.283	0.0464	*
Fusarium sp._*13*	0.635	0.0131	*
Fusarium sp._*15*	0.422	0.0136	*
Fusarium sp._*17*	0.319	0.0288	*
Fusarium sp._*2*	0.289	0.0136	*
Fusarium sp._*6*	0.421	0.0015	**
Lophiostoma sp.*	0.759	0.0004	***
Lophiostoma sp._*10**	0.636	0.0026	**
Lophiostoma sp._*11**	0.686	0.0001	***
Minutisphaera aspera	0.697	0.0125	*
Minutisphaera sp._*1*	0.403	0.0041	**
Phaeosphaeria sp._*2*	0.335	0.0259	*
Phoma sp._*1* †	0.351	0.0335	*
Symmetrospora symmetrica	0.275	0.0271	*
Thelonectria olida †	0.453	0.001	***
Thelonectria sp. †	0.548	0.0006	***
unknown_107	0.46	0.0215	*
unknown_119	0.363	0.002	**
unknown_16	0.403	0.0139	*
unknown_28	0.42	0.0073	**
unknown_38	0.417	0.0006	***
unknown_4	0.413	0.0208	*
unknown_45	0.427	0.0293	*
unknown_6	0.376	0.0123	*
unknown_62	0.405	0.0301	*
unknown_96	0.533	0.0003	***

Table S3. Indicator species for control and HWT plants in **(a)** Nursery I and **(b)** Nursery II. Nurseries were analyzed separately. Correlation values (stat) and the statistical significance of the correlation (p-values) are reported. GTD-related indicator species are marked in bold and biocontrol species are marked with an asterisk (*).

a. Nursery I

7 species associated with control plants

**Indicator species**	**stat**	***p* value**	**significance**
Cadophora luteo-olivacea_2 †	0.854	0.0001	***
Cadophora sp. †	0.766	0.0008	***

Cortinarius sp.	0.483	0.0339	*
Diaporthe sp.*_1* †	0.782	0.0025	**
Lophiostoma sp.*	0.847	0.0004	***
Niesslia sp.*_4*	0.575	0.0152	*
Stagonospora sp.*_4*	0.65	0.0072	**
**10 species associated with HWT plants**			
Acremonium sp.*_13* †	0.818	0.0012	**
Acremonium sp.*_15* †	0.771	0.0028	**
Alternaria sp.*_6**	0.767	0.0289	*
Cystobasidium lysinophilum	0.789	0.0009	***
Cystobasidium slooffiae_1	0.692	0.0012	**
Fusarium sp.*_22*	0.888	0.0001	***
Fusarium sp.*_3*	0.923	0.0001	***
Fusarium sp.*_6*	0.853	0.0001	***
Stagonospora sp.*_5*	0.636	0.0372	*

b. Nursery II

**Nine species associated with control plants**			
**Indicator species**	**stat**	***p* value**	**significance**
Acaromyces ingoldii	0.769	0.002	**
Cadophora luteo-olivacea_2 †	0.797	0.0236	*
Cadophora sp. †	0.682	0.0474	*
Diaporthe sp.*_1* †	0.733	0.0084	**
Niesslia sp.*_4*	0.679	0.0152	*
Ophiobolus sp.	0.656	0.0012	**
Stagonospora sp.*_4*	0.5	0.0257	*
unknown_53	0.777	0.0011	**
unknown_98	0.689	0.0156	*
**Five species associated with HWT plants**			
Acremonium sp.*_13* †	0.779	0.0486	*
Cystobasidium lysinophilum	0.743	0.0469	*
Cystobasidium slooffiae_1	0.757	0.0092	**
Fusarium sp.*_3*	0.842	0.0011	**
Fusarium sp.*_6*	0.649	0.0012	**

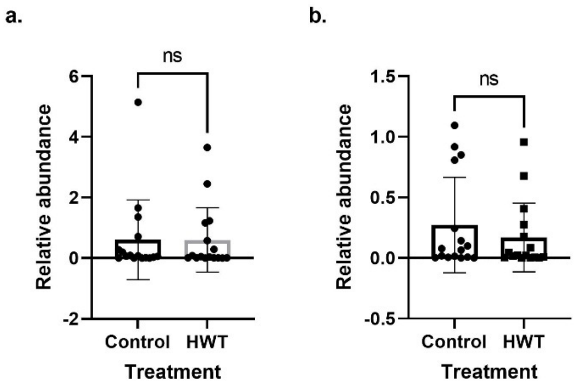

Figure S1. Relative abundance of GTD-related fungi in control and HWT-treated plants in (a) graft unions and (b) root collars.
